# FGFR3 gene mutation plus GRB10 gene duplication in a patient with achondroplasia plus growth delay with prenatal onset

**DOI:** 10.1186/s13023-016-0465-4

**Published:** 2016-07-02

**Authors:** Haiming Yuan, Linhuan Huang, Xizi Hu, Qian Li, Xiaofang Sun, Yingjun Xie, Shu Kong, Xiaoman Wang

**Affiliations:** Guangzhou KingMed Center for Clinical Laboratory Co., Ltd, Guangzhou, 510330 Guangdong China; KingMed School of Laboratory Medicine, Guangzhou Medical University, Guangzhou, 510330 Guangdong China; Fetal Medicine Centre, Department of Obstetrics and Gynaecology, The First Affiliated Hospital of Sun Yat Sen University, Guangzhou, Guangdong 510080 China; Fairmont Preparatory Academy, Anaheim, CA 92801 USA; Affymetrix Biotech Shanghai Ltd., Shanghai, 200020 China; Key Laboratory for Major Obstetric Diseases of Guangdong Province, Key Laboratory of Reproduction and Genetics of Guangdong Higher Education Institutes, The Third Affiliated Hospital of Guangzhou Medical University, 63 Duobao Rd., Guangzhou, 510150 People’s Republic of China

**Keywords:** Achondroplasia, *GRB10*, Duplication, Over-expression, Silver-Russell syndrome

## Abstract

**Background:**

Achondroplasia is a well-defined and common bone dysplasia. Genotype- and phenotype-level correlations have been found between the clinical symptoms of achondroplasia and achondroplasia-specific * FGFR3* mutations.

**Result:**

A 2-year-old boy with clinical features consistent with achondroplasia and Silver-Russell syndrome-like symptoms was found to carry a mutation in the fibroblast growth factor receptor-3 (FGFR3) gene at c.1138G > A (p.Gly380Arg) and a *de novo* 574 kb duplication at chromosome 7p12.1 that involved the entire growth-factor receptor bound protein 10 (GRB10) gene. Using quantitative real-time PCR analysis, *GRB10* was over-expressed, and, using enzyme-linked immunosorbent assays for *IGF1* and IGF-binding protein-3 (IGFBP3), we found that *IGF1* and *IGFBP3* were low-expressed in this patient.

**Conclusions:**

We demonstrate that a combination of uncommon, rare and exceptional molecular defects related to the molecular bases of particular birth defects can be analyzed and diagnosed to potentially explain the observed variability in the combination of molecular defects.

**Electronic supplementary material:**

The online version of this article (doi:10.1186/s13023-016-0465-4) contains supplementary material, which is available to authorized users.

## Background

Achondroplasia (ACH, OMIM #100800) is the most common non-lethal skeletal dysplasia. It is an autosomal-dominant disorder with an incidence of approximately 5–15 per 100,000 live births. Mutations in *FGFR3* at c.1138 G > A (p.Gly380Arg) are known to cause ACH, with 97 % of cases involving this mutation [8, 22]. Gain-of-function mutations in *FGFR3* have been shown to cause both chondrodysplasias and craniosynostoses and to result in impaired endochondral ossification [3].

While patients with maternal uniparental disomy of chromosome 7 (mUPD7) that involves *GRB10* are consistently diagnosed with Silver–Russell syndrome (SRS, OMIM #180860) [4, 7]. SRS is a rare congenital developmental disorder that is characterized by severe intrauterine and postnatal growth restriction, a triangular-shaped face, and congenital malformations that include relative macrocephaly, hemihypotrophy, and fifth-finger clinodactyly [24]. However, there is no conclusive evidence regarding the pathogenic mechanism by which GRB10 (i.e., imprinting vs. dosage) contributes to the etiology of SRS.

Currently, only 6 *SRS* patients have been shown to carry *GRB10* duplications. In 1999, Joyce et al. described a mother and daughter who were both diagnosed with SRS and who both carried a duplication at 7p12.1-p13, which includes *GRB10* and *IGFBP1*. The mother carried a paternally derived duplication, and the daughter carried a maternally derived duplication [13]. The next year, Monk et al. reported a *de novo* maternal duplication of 7p11.2-p13 in a patient with *SRS* [17]. Subsequently, two more individuals (A.C. and H.C.) with *SRS* were reported to possess larger duplications at this same locus: A.C. carried a maternally derived duplication, but the parent of origin for H.C.’s duplication was not known [16]. Recently, Eggermann reported a boy who displayed heterogeneous growth patterns and carried a *de novo* paternally derived duplication at 7p12.2 [6]. A few additional cases have been reported to the Decipher and ISCA databases (Fig. 4a, Table 1). All of these reported duplications involved the *GRB10* gene, which is regarded as the most promising candidate gene at this locus (Fig. 2). Because all of these duplications were large (except Decipher case 289205, which had a partial *GRB10* gene duplication), it is possible that neighboring genes, particularly genes such as *IGFBP1* and *IGFBP3*, could contribute to SRS, and the involvement of these genes has not been completely ruled out in these cases. As a result, the correlations between SRS genotypes and phenotypes that are associated with duplications of *GRB10* remain incompletely understood.

**Table 1:** Review genomic and clinical information on patients with duplication involving *GRB10*

Patient/source	Our patient	Patients TB/LB from Joyce et al. [13]	Patient DP from Monk et al. [17]	Patients HC/AC from Monk et al. [16]	Patient from Eggermann et al. [6] (decipher 285981)	Decipher 289205	Decipher 276327	ISCA nssv579045
Genomic location (hg19)	chr7:50654827-5122921	chr7:42029531–51138357 (D7S691-D7S242)	chr7:40742989–50861159 (D7S1769-GRB10)	chr7:41392336–54000000 (RP5-953B5-7p11.2)	chr7:47034222–52175639	chr7:50713413-50982532	chr7:43566637-51282735	chr7:33367924-61831899
Duplication size	574Kb	~10 cM	~10 cM	>10 cM	5.1 Mb	270Kb	7.72 Mb	28.5 Mb
Inheritance/orign	*de novo*/maternal	LB: maternal	*de novo/*maternal	AC: maternal	de novo/paternal	inherited/paternal	*de novo*/unknown	unknown
		TB: paternal		HC: unknown				
Phenotype	SRS	SRS	SRS	SRS-like	overgrowth, DD, microcephaly, seizure	hypotonia, GDD	muscular hypotonia	FTT, GDD, large eyes, microcephaly, short stature, triangular face
Detection method	array	karyotype/FISH	karyotype/FISH	karyotype/FISH	array	array	array	array
Genes involved (Refseq)	*GRB10* and part of *COBL*	>50 genes	>50 genes	>50 genes	>20 genes	Part of *GRB10*	>40 genes	>180 genes

*Abbreviation: FISH* fluorescence *in situ* hybridization, *DD* developmental delay, *GDD* Global developmental delay, FTT: failure to thrive

Here, we report a patient who displayed prenatal onset growth delay and ACH and carried a small *de novo* duplication at 7p12.1 that involved the *GRB10* gene in addition to a heterozygous point mutation at *FGFR3*. We detected the smallest known duplication in a patient who displayed one of the typical clinical features of SRS. This duplication was restricted to the *GRB10* gene at 7p12.1. This case provides direct evidence showing that a *GRB10* duplication is sufficient to cause the prenatal onset growth delay phenotype in SRS and the combination of these two molecular defects may explain the so-called variable expressivity dominant traits.

## Methods

### Clinical report

**Fig. 1:**
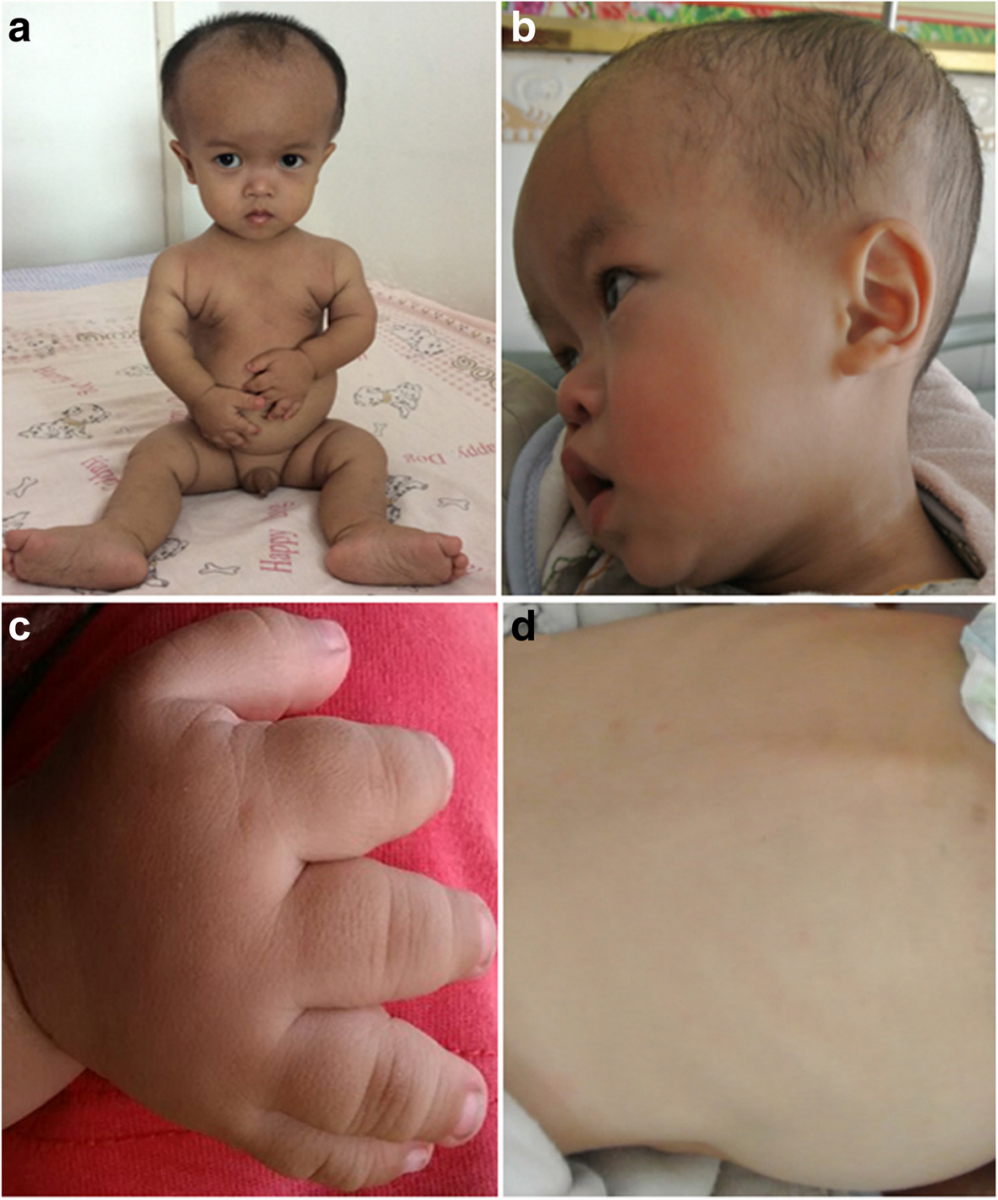
Clinical features of the proband at 2 years of age. **a** Note the prominent forehead, triangular face, downward-slanting mouth and micrognathia. The proband also presented with very short limbs and body asymmetry, **b** a low nasal bridge, and **c** trident hands and fifth finger clinodactyly, **d** scoliosis

A 2-year-old Chinese boy was referred and admitted to our hospital because of congenital malformations and development delay over the previous month. He presented with a characteristically small triangular face with a prominent forehead and low nasal bridge, micrognathia and downturned corners of the mouth, and sparse hair (Fig. 1). He could not walk but could sit with support, and his language development was significantly delayed. He had been previously diagnosed with global developmental delay (date not provided), and he exhibited short limbs and relative macrocephaly without compensatory growth at 6 months of age.

**Fig. 2:**
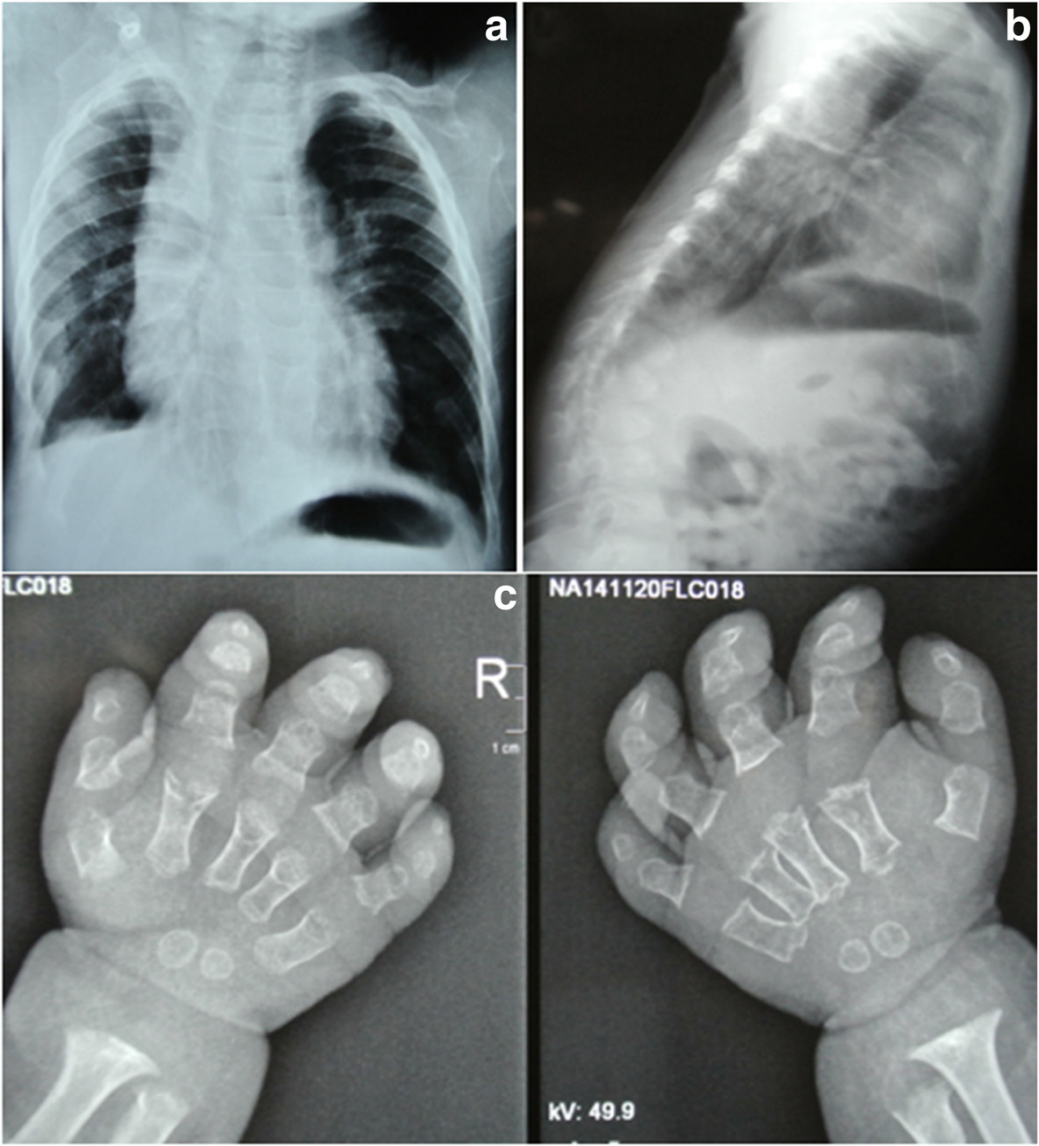
Spine X-ray images showed mild kyphosis (**a**) and scoliosis (**b**) at the thoracic and lumbar region. Bone age is delayed (**c**)

**Fig. 3:**
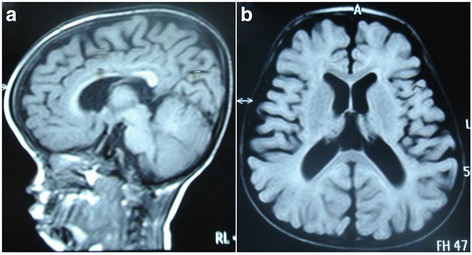
Brain MRI images showed a hypoplastic corpus callosum, enlargement of the lateral ventricles and hydrocephalus (**a** and **b**)

A physical examination resulted in the following findings: height, 66 cm (<1 rd percentile); weight, 7.5 kg (<1 rd percentile); head circumference, 52 cm (<3 rd percentile); and BMI, 17.2 kg/m^2^, indicating a persistent failure to thrive. In addition, the patient had trident hands and fifth finger clinodactyly. An X-ray of the hand showed stubby fingers, metaphyseal enlargement, bilateral slight curvature of the radius and ulna, and delayed bone age. A spine X-ray showed mild scoliosis and kyphosis at the thoracic and lumbar region (Fig. 2), which indicated achondroplasia. A cerebral MRI showed a hypoplastic corpus callosum, enlargement of the lateral ventricles and hydrocephalus (Fig. 3). In addition, he exhibited muscular hypotonia and sleep-related symptoms (e.g., snoring, mouth breathing, observed apneas, and excessive sweating). An ophthalmological examination and neurometabolic investigation were normal. Based on the above clinical phenotype, a diagnosis of SRS-like symptoms and achondroplasia was suspected.

The patient was the fourth child of healthy unrelated parents with a negative family history. His siblings were all healthy. Fetal short limbs were noticed on the ultrasound examination at 8 months of pregnancy, indicating intrauterine growth restriction (date not provided). He was born by vaginal delivery at 38 weeks of gestation after an uneventful pregnancy. His birth weight was 1900 g (<1st percentile), his length was 43 cm (<1st percentile) and his head circumference was 34 cm. His Apgar scores were all 9 s. Feeding difficulty was noted during the first two months after birth.

### Karyotyping and chromosomal microarray analysis

A standard chromosome analysis at 550-band resolution was performed using cultured peripheral blood from the patient according to standard procedures.

A chromosomal microarray analysis (CMA) of the patient and his parents was performed using an Affymetrix Cytoscan HD Array (Affymetrix, Santa Clara, CA, USA). Genomic DNA was extracted from peripheral blood using a commercial kit (Qiagen). The labeling and hybridization procedures were performed according to the manufacturer’s instructions. The raw CMA data were analyzed using Affymetrix Chromosome Analysis Suite software. The microarray data for the family trio were then processed using the standard protocols provided by the manufacturer to perform a basic analysis using Chromosome Analysis Suite software (NetAffx 33.1, Affymetrix, USA).

### Confirmation of GRB10 duplication

The GRB10 copy number was further confirmed using quantitative real-time PCR analysis. The primer sequences that were used and their descriptions are included in Additional file 1: Table S1.

### GRB10 expression level

*GRB10* expression levels were detected in blood samples using quantitative real-time PCR analysis. Total RNA was extracted from whole blood, and reverse transcription was performed using a commercial kit (Promega, USA). A blood sample from the proband’s mother was used as the control. The primer sequences that were used and their descriptions are included in Additional file 1: Table S2.

### Measurement of IGF1 and IGFBP3

We measured the levels of *IGF1* and *IGF* binding protein-3 (IGFBP3) in the patient’s serum using enzyme-linked immunosorbent assay (ELISA) kits (Diagnostic Systems Laboratories, Webster, TX, USA) according to the manufacturer’s instructions.

### Sequencing of the fibroblast growth factor receptor-3 gene (FGFR3)

DNA samples were tested for common disease-associated mutations in *FGFR3* (achondroplasia and hypochondroplasia: p.Gly380Arg and p.Asn540Lys, respectively; thanatophoric dysplasia type I: p.Arg248Cys, p.Tyr373Cys, p.Ser249Cys, and p.X807; and thanatophoric dysplasia type II: p.Lys650Glu) using Sanger sequencing. The primer sequences are shown in Additional file 1: Table S3 (reference sequence for the *FGFR3* gene: NM_000142.4). Sanger sequencing was performed according to the manufacturer’s instructions using a Big Dye Terminator Cycle Sequencing Kit (version 3.1) and analyzed using an ABI Prism 3130xl Genetic DNA analyzer.

## Results

**Fig. 4:**
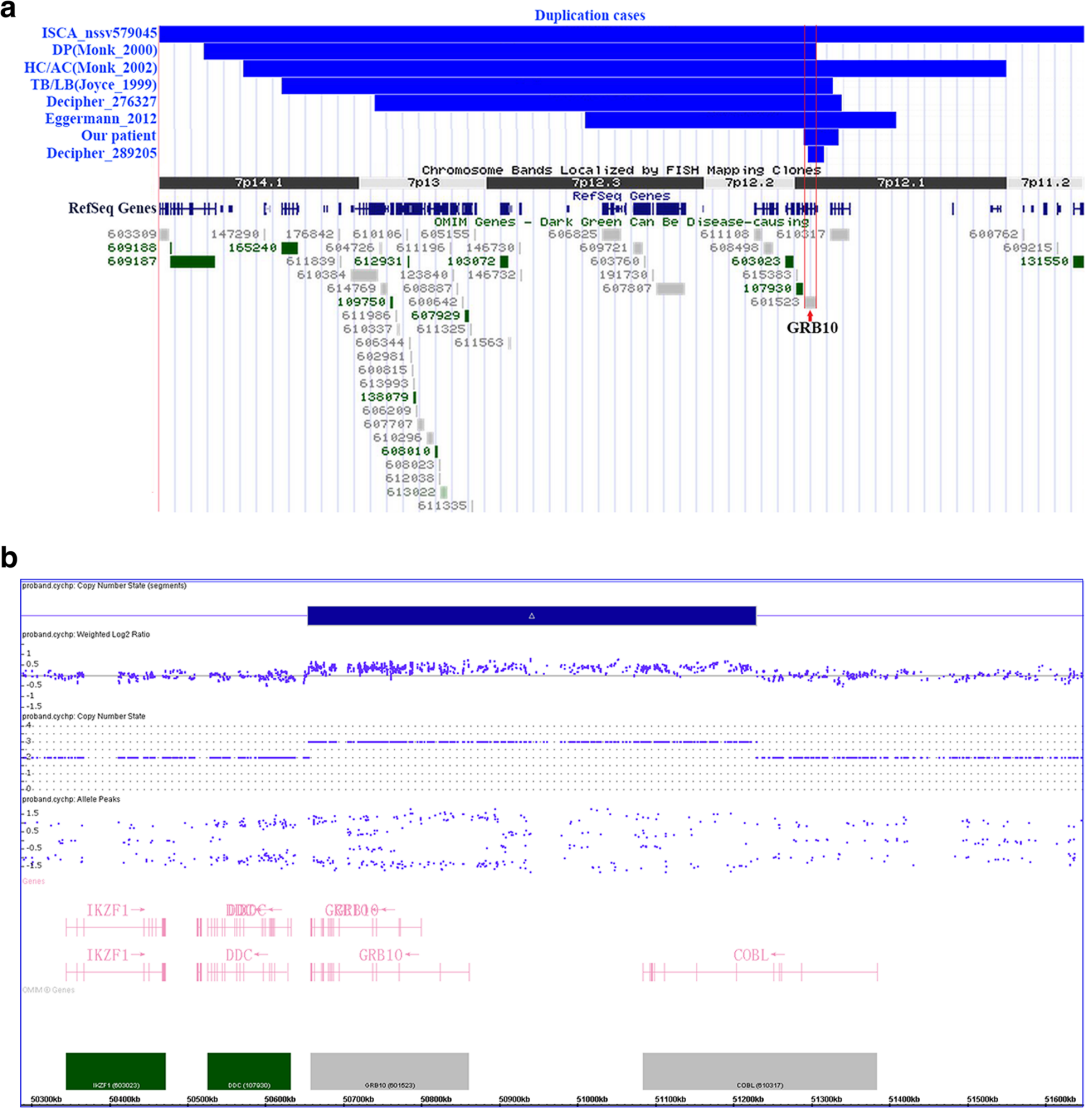
Overview of previously described cases shown to have genomic imbalances at 7p12.1 that involved *GRB10*. (Blue: duplications). Red vertical lines demarcate the genomic interval of the *GRB10* gene (**a**). Scatter plot of a Cytoscan HD array at 7p12.1 showing a duplication interval involving the whole GRB10 gene and part of the COBL gene (**b**)

**Fig. 5:**
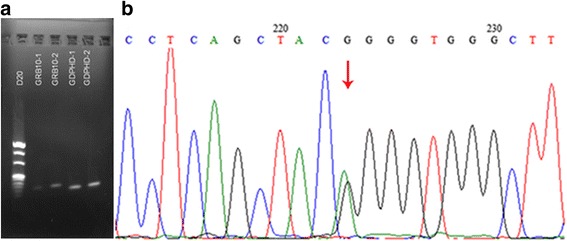
**a** *GRB10* expression levels were analyzed in blood samples using RT-PCR. **b** DNA sequences corresponding to the patient’s *FGFR* genes. A FGFR3 c.1138 G > A (p.Gly380Arg) mutation was found

Karyotyping analysis of the patient revealed a normal 46, XY karyotype. CMA revealed a 574 kb duplication at 7p12.1 (arr[hg19] 7p12.1(50,654,827- 51,229,221) × 3dn) (Fig. 4). This duplication was not detected in either the patient’s parents or siblings, indicating that it was a *de novo* duplication. We used Chromosome Analysis Suite software (NetAffx 33.1, Affymetrix, USA) to analyze the family trio SNP-array data, and we found that the duplication originated from the mother. The duplication was further confirmed using RT-PCR analysis. The *GRB10* expression level in the patient’s blood was three-fold higher than the level in his mother’s blood (Fig. 5a). The levels of *IGF1* and *IGFBP3* in the patient were <25.0 ng/ml and 1.78 ng/ml, respectively, which was far below normal levels. Sequencing of the *FGFR3* gene identified a *FGFR3* c.1138 G > A (p.Gly380Arg) mutation (Fig. 5b).

## Discussion

The patient exhibited features such as scoliosis and a trident configuration of the hands all of which can be explained by a mutations in *FGFR3* at c.1138 G > A(p.Gly380Arg). However, the speech delay, hypotonia and small triangular face phenotypes were not commonly reported in previous cases of ACH (Table 2) [9, 12]. A hypoplastic corpus callosum and hydrocephalus could potentially be CNS manifestations of non-treated/severe achondroplasia. In addition, the observation of a global developmental delay could not be definitively separated from the consequences of the identified *FGFR3* mutation. Finally, at birth, patients with achondroplasia present with a relatively short stature that does not affect weight, whereas this patient had a low weight at birth. In addition, no overt body asymmetry was reported in this patient. Accordingly, the one unique feature of this patient that can be directly linked to a *GRB10* duplication is the prenatal onset growth delay.

**Table 2:** Comparison of clinical abnormalities with Achondroplasia (ACH) and Silver-Russell syndrome (SRS)

Clinical abnormalities	Achondroplasia (ACH)	Silver-Russell syndrome (SRS)	Our patient
Prenatal IUGR	-	+	+
Special face			
macrocephaly	-	+	+
broad foreheads	+	-	+
pointed	-	+	+
triangular shaped	-	+	+
nose bridge collapse	+	-	+
small chins	-	+	+
Hand			
a trident configuration of the hands	+	-	+
fifth finger clinodactyly	-	+	+
Body			
asymmetry of limbs	-	+	+
vertebrae	scoliosis	thoracic kyphosis	Scoliosis and thoracic kyphosis
Hypotonia	-	+	+
Developmental delay	-	+	+
Delayed speech	-	+	+
Intellectual disability	-	+	+
Feeding difficulties in early childhood	-	+	+
Snoring (coarse breath sounds)	+	-	+

In our patient, we detected a three-fold increase in *GRB10* expression. *GRB10* is an imprinted gene on 7p12.1. Because it is a key genetic component of developmental programming, recent studies have also suggested that placental *GRB10* plays a role in regulating fetoplacental growth and that it is thereby implicated in the pathophysiology of fetal growth restriction in the context of fetal gender [2, 26]. Studies have consistently supported the notion that increases in gene dosage are the main contributors to the over-production of the *GRB10* gene product. The *GRB10* gene encodes an adaptor protein that interacts with *IGF1* and insulin receptors to negatively regulate the *IGF1* growth cascade [5, 18]. A previous study reported that endogenous *GRB10* expression was increased in the hippocampus of rats with diabetic encephalopathy and that this increase might result in damaged nerve functions and cognitive impairments. In addition, the insulin/IGF1 signaling pathways have been implicated in dysregulated synaptic maturation and may play key roles in brain aging and dementia as well as learning and cognitive functions in rodent models [14, 19]. In the present study, the *IGF1* and *IGFBP3* levels in the patient were far below normal levels, consistent with a model in which the duplication of *GRB10* directly inhibits the phosphorylation of the *IGF1R* substrate and reduces the level of *Grb10* expression to below normal limits, which may result in nervous system impairments such as growth delays and intellectual disabilities (ID) [23]. These findings suggest that the duplication of *GRB10* contributes to the prenatal onset growth delay phenotype that was observed in our patient and may also explain the ID features in SRS.

Previous studies of mice with uniparental disomy of Grb10 demonstrated that Grb10 is a typical imprinted gene and that it affects growth mainly via an imprinting mechanism [1, 20, 21, 25]. However, the imprinting behavior of human *GRB10*, which is located on chromosome 7, has been shown to be different from that of its mouse homolog [11]. Human *GRB10* is biallelically expressed in most tissues, with the exception of the placental villus trophoblast and fetal brain tissues [10, 15]. Because of the known complexity of the 7p12.1 imprinting regions and their interactions, interpretations of copy number variations in this region are complicated. The clinical outcomes in cases of micro-duplications are influenced by the size, breakpoint positions and parental inheritance of the imbalance and by the imprinting status of the affected genes.

Though, the duplication that was identified in our propositus could potentially have disrupted the genomic structure of the cordon-bleu WH2 repeat protein (COBL) gene, which is specifically expressed in the node and its derivatives until organogenesis, we could not exclude the possibility that a disruption in *COBL* might have been involved in the patient’s phenotype. In addition, there are no known correlations between mutations in *FGFR3* and the duplication of *GRB10*.

## Conclusion

The data related to the patient described in the present study at least suggest that mutations in *FGFR3* cause ACH but do not influence the effects of the duplication of *GRB10* on prenatal onset growth delay in *SRS*. The results of our study also suggest that phenotypes are rarely “simple” or directly related to specific gene defects and that combinations of uncommon, rare and exceptional molecular defects, which can be explored and used in diagnoses, may explain the so-called variability observed in the expression of dominant traits.

## Abbreviation

CMA, chromosomal microarray analysis; COBL, cordon-bleu WH2 repeat protein; ELISA, enzyme-linked immunosorbent assay; FGFR3, fibroblast growth factor receptor-3; FGFR3, fibroblast growth factor receptor-3; GRB10, growth-factor receptor-bound protein 10; H19DMR, H19 promoter; HMGA2, high-mobility AT-hook 2; ICR1, Imprinted Center Region 1; ID, intellectual disability; IGFBP3, IGF binding protein-3; mUPD7, maternal uniparental disomy of chromosome 7; SRS, Silver–Russell syndrome

## Additional file

Additional file 1: Supplemental Tables S1, S2 and S3. (XLSX 16 kb)
